# Identification of two unannotated miRNAs in classic Hodgkin lymphoma cell lines

**DOI:** 10.1371/journal.pone.0283186

**Published:** 2023-03-24

**Authors:** Adam Ustaszewski, Julia Paczkowska, Joanna Janiszewska, Stephan H. Bernhart, Julia Bein, Núria Russiñol, Martin-Leo Hansmann, Vicente Chapaprieta, José I. Martín-Subero, Reiner Siebert, Sylvia Hartmann, Maciej Giefing

**Affiliations:** 1 Institute of Human Genetics, Polish Academy of Sciences Poznan, Poznan, Poland; 2 Interdisciplinary Center for Bioinformatics, Transcriptome Bioinformatics, University of Leipzig, Leipzig, Germany; 3 Dr. Senckenberg Institute of Pathology, Goethe University Frankfurt, Frankfurt am Main, Germany; 4 Institut d’Investigacions Biomèdiques August Pi I Sunyer, IDIBAPS, Barcelona, Spain; 5 Frankfurt Institute of Advanced Studies, Frankfurt am Main, Germany; 6 Institute of General Pharmacology and Toxicology, Goethe University Frankfurt, Frankfurt am Main, Germany; 7 Centro de Investigación Biomédica en Red de Cáncer (CIBERONC), Pamplona, Spain; 8 Facultat de Medicina, Hospital Clínic de Barcelona and Departament de Fonaments Clínics, Universitat de Barcelona, Barcelona, Spain; 9 Institució Catalana de Recerca i Estudis Avançats, ICREA, Barcelona, Spain; 10 Institute of Human Genetics, Ulm University and Ulm University Medical Center, Ulm, Germany; The University of Texas MD Anderson Cancer Center, UNITED STATES

## Abstract

MicroRNAs (miRNAs) are small non coding RNAs responsible for posttranscriptional regulation of gene expression. Even though almost 2000 precursors have been described so far, additional miRNAs are still being discovered in normal as well as malignant cells. Alike protein coding genes, miRNAs may acquire oncogenic properties in consequence of altered expression or presence of gain or loss of function mutations. In this study we mined datasets from miRNA expression profiling (miRNA-seq) of 7 classic Hodgkin Lymphoma (cHL) cell lines, 10 non-Hodgkin lymphoma (NHL) cell lines and 56 samples of germinal center derived B-cell lymphomas. Our aim was to discover potential novel cHL oncomiRs not reported in miRBase (release 22.1) and expressed in cHL cell lines but no other B-cell lymphomas. We identified six such miRNA candidates in cHL cell lines and verified the expression of two of them encoded at chr2:212678788–212678849 and chr5:168090507–168090561 (GRCh38). Interestingly, we showed that one of the validated miRNAs (located in an intron of the *TENM2* gene) is expressed together with its host gene. *TENM2 is* characterized by hypomethylation and open chromatin around its TSS in cHL cell lines in contrast to NHL cell lines and germinal centre B-cells respectively. It indicates an epigenetic mechanism responsible for aberrant expression of both, the *TENM2* gene and the novel miRNA in cHL cell lines. Despite the GO analysis performed with the input of the *in silico* predicted novel miRNA target genes did not reveal ontologies typically associated with cHL pathogenesis, it pointed to several interesting candidates involved in i.e. lymphopoiesis. These include the lymphoma related *BCL11A* gene, the *IKZF2* gene involved in lymphocyte development or the transcription initiator *GTF2H1*.

## Introduction

MicroRNAs (miRNAs) play a key role in posttranscriptional regulation of gene expression, and are essential regulators of various cellular processes [1,2]. These short RNAs incorporated into the RISC complex bind to the complementary 3’UTR fragment of a target mRNA leading to its degradation or inhibition of translation [3]. Aberrant miRNA expression in classic Hodgkin lymphoma (cHL), a B-cell lymphoma characterized by the presence of few large neoplastic Hodgkin and Reed-Sternberg (HRS) cells, was first confirmed in the early 2000s [4]. By now, the role of several oncomiRs and tumor suppressor miRNAs was described in cHL [5–7]. These include the deregulated miR-155 and miR-196a and miR-23a-3p, which are assumed to contribute to the constitutive NFκB hyperactivation, a process crucial in cHL pathogenesis [6,8,9]. Moreover, also other miRNAs deregulated in cHL such as miR-9, miR-138, miR-150 were described as candidates involved in processes responsible for Hodgkin lymphomagenesis such as immune evasion and impaired B-cell receptor signalling [9].

In our recent study we have performed high throughput screening of miRNA expression in 7 widely used cHL cell lines and ten non-Hodgkin lymphoma cell lines (NHL) by RNA-seq [9]. Here we mined this dataset with the aim to find miRNAs expressed in cHL and not yet reported in miRBase (release 22.1). This brought us to the identification of two molecules with typical miRNA characteristics which are recurrently expressed in cHL cell lines. Moreover, our results suggest that the aberrant expression of these novel miRNAs is at least to some extend caused by the loss of epigenetic control in cHL cell lines.

## Materials and methods

### CHL cell lines, NHL cell lines and primary cHL samples

7 cHL cell lines (L-428 [10], HDLM-2 [11], KM-H2 [12], L-1236 [13], SUP-HD1 [14], U-HO1 [15], L-540 [16]) and 10 NHL cell lines (Burkitt lymphoma: Raji [17], Ca46 [18], Daudi [19], Namalwa [20], Ramos [21]; Diffuse Large Cell Lymphoma: OCI-LY1 [22], OCI-LY3 [22], OCI-LY7 [22], SU-DHL-6 [23]; B Cell Lymphoma: Val [24]) were used. Three cHL cell lines (HDLM-2, SUP-HD1, U-HO1) and ten NHL cell lines (Burkitt lymphoma: Raji, Ca46, Daudi, Namalwa, Ramos; Diffuse Large Cell Lymphoma: OCI-LY1, OCI-LY3, OCI-LY7, SU-DHL-6; B Cell Lymphoma: Val) were obtained from DSMZ GmbH (Braunschweig, Germany). The L-428, KM-H2, L-1236, L-540 cHL cell lines were obtained from collaborating partners and together with HEK-293 cell line were STR typed to confirm their identity. Furthermore, the HEK-293 cell line was used for the transfection experiments in functional analysis [9]. Respective cell culture conditions were described previously [25].

Normal CD77^+^ GC (germinal centre) B cells were purified from remnants of fresh tonsils of routine tonsillectomy samples using magnetic activated cell sorting (MACS; Miltenyi Biotech, Bergisch Gladbach, Germany), as reported previously [26]. Primary HRS cells and negative controls (empty membrane fragments adjacent to lymph node sections) were obtained by laser microdissection (PALM Robot MicroBeam laser microdissection system, PALM) from HE stained lymph node sections. 1000 microdissected cells were obtained from each patient and used in further analyses. The local ethics committee of Goethe University Hospital (157/17) approved the study and informed consent from the patients was obtained in accordance with the Declaration of Helsinki. The informed consent from the patients was written and all participants were adults.

### Small RNA profiling (miRNA-seq)

The BAM files from miRNA-seq of seven cHL cell lines and 10 NHL cell lines [9] were reanalysed in order to detect unannotated RNA-like sequences as potential miRNA candidates. For further identification of putative miRNAs we used the CAP-miRSeq software (http://bioinformatics.mayo.edu/research/cap-mirseq/). miRNA candidates were compared to known, annotated miRNAs from miRBase (release 22.1) [27] and selected according to structural properties characteristic for miRNA sequences.

To identify potential miRNAs expressed in cHL we used the following criteria:

- Not annotated miRNA expressed in at least 3/7 of the analyzed cHL cell lines and absent in NHL cell lines (“expressed” defined as mean ≥ 20 CPM (Counts Per Million) in cell lines with expression).
- Not annotated miRNAs expressed in less than 2% of the analyzed samples from previously published miRnome sequencing data including 56 primary germinal center derived B-cell lymphomas (16 Burkitt lymphomas, 19 diffuse large B-cell lymphomas, and 21 follicular lymphomas recently analysed within the International Cancer Genome Consortium Project "Determining Molecular Mechanisms in Malignant Lymphoma by Sequencing" (ICGC MMML-Seq) [28]
- Manual curation in order to exclude known miRNAs, palindromic sequences, artefacts and other small ncRNAs.

### RNA isolation and real-time qPCR analyses

cDNA templates for RT-qPCR we obtained by reverse transcription of 10 ng of total RNA (cHL cell lines and germinal center B-cells—GCB) and 10 μl of miRNA containing eluate (HRS cells obtained as described by Küppers et al. [29]) using TaqMan^TM^ Advanced miRNA cDNA Synthesis Kit (Thermo Fisher Scientific) [30] according to manufacturer’s protocol. Real-time qPCR was performed using custom TaqMan™ probes corresponding to the mature miRNA sequence for each of the selected candidate miRNA (**S1 Table**). Relative expression of selected miRNAs was calculated using BioRad Genex software [31] in relation to reference miRNAs (miR-191-5p and miR-361-5) as described previously [25].

For mRNA expression analysis total RNA (500 ng) isolated from the cell lines using Trizol [32] was reverse transcribed using Maxima First Strand cDNA Synthesis Kit (Thermo Scientific, USA) as described in the manufacturer’s protocol. Primers were designed using Primer 3 Plus software (**S2 Table**). PCR reactions were performed as described previously [33]. The results were analysed using BioRad Genex software in respect to the *ACTB* and *GAPDH* reference genes. The BioRad Genex software determines particular cycles (Ct) using automatically generated background values. Next, for each sample, it calculates the quantity relative to control genes, taking into account the amplification efficiency of qPCR for each gene [31].

### miRNA gene cloning and transfection

Selected novel miRNA genes were amplified with 100 bp flanks using primers designed with the Primer 3 Plus software (**S3 Table**). PCR products were cloned into the pCDH-CMV-MCS-EF1α-Green Puro cloning vector (SBI, Palo Alto, CA, USA) and used for transformation of JM109 *E*.*coli* competent cells (Promega) as described previously [34]. The plasmid DNA (pDNA) was isolated using PhasePrep^TM^ BAC DNA kit (Sigma‐Aldrich, St. Louis, MO, USA). For transfection of the HEK-293 cell line the Lipofectamine RNAiMAX Transfection Reagent (Invitrogen, Karlsruhe, Germany) was used according to the manufacturer’s protocol. For control purposes the same cell line was transfected using the empty vector. Each transfection was performed in two biological replicates. Two days past transfection, total RNA was isolated using Trizol. Reverse transcription and real-time qPCR reactions were performed as described above. The transfection efficiency of HEK-293 cells was measured using JuLi Br & FL Station (NanoEnTek, Seoul, Korea) by the calculation of GFP positive cells. RT-qPCR miRNA expression from the construct was measured using same TaqMan^TM^ probes as described above in reference to hsa-miR-423-3p **(S1 Table)** [25].

### DNA isolation and bisulfite DNA pyrosequencing

DNA from cell lines was isolated using phenol/chloroform and Phase Lock Gel™ tubes (5Prime Quantabio, Beverly, MA, USA) followed by ethanol precipitation and used for bisulfite conversion.

DNA was bisulfite converted using the EpiTect DNA Modification Kit (Qiagen, Hilden, Germany) according to the manufacturer’s protocol. PCR reactions were prepared using PyroMark PCR Kit (Qiagen) in a DNA/RNA UV-cleaner box UVC/T-AR (Biosan, Riga, Latvia). The PCR reaction mixture contained: 12.5 μl PyroMark Master Mix; 0.5 μl (20 pmol/μl) of F and R primer, 2.4 μl CoralLoad, 1 μl of converted DNA (~25 ng/μl) and 8 μl of RNase-Free water. PCR primers (assays) (**S4 Table**) designed using the PyroMark Assay Design 2.0 software (Qiagen, Germany) were used for DNA methylation analysis of three regions corresponding to the two novel miRNA candidates:

1. 2_nv_chr2_212678788 novel miRNA candidate:
  - region (I) located 54 bp upstream of the miRNA gene, amplified sequence: chr2:212,678,797–212,679,018 (GRCh38/hg38), genomic position of analysed dinucleotides: chr2:212,678,922; chr2:212,678,938; chr2:212,678,945 (GRCh38/hg38).
2. 3_nv_chr5_168090507 novel miRNA candidate:
  - Region (I) 166 bp upstream of *TENM2* (transcript uc010jjd.3, putative novel miRNA primary transcript, amplified sequence: chr5:167,284,564–167,284,717 (GRCh38/hg38), the genomic position of analysed dinucleotide: chr5:167,284,682 (GRCh38/hg38),
  - Region (II); ~2 kb upstream of the miRNA gene, amplified sequence: chr5:168,088,403–168,088,609 (GRCh38/hg38), genomic position of analysed dinucleotides: chr5:168,088,484; chr5:168,088,488; chr5:168,088,497; chr5:168,088,504; chr5:168,088,522; chr5:168,088,531; chr5:168,088,535 (GRCh38/hg38).

PCR reactions were performed in the following conditions: 95°C for 15 min × 1; 94°C for 30 s, 59°C for 30 s, 72°C for 30 s × 45; 72°C for 10 min × 1; 4°C ∞. The products were visualised on 1.8% agarose gel with SimplySafe™ (EURx) under UV light (BioDoc‐it Imaging System, UVP, USA). Purification of PCR products and pyrosequencing was performed as described previously [35]. Each pyrosequencing experiment was performed with fully methylated DNA control (CpG Genome Universal Millipore, Darmstadt, Germany) and unmethylated whole genome amplified (WGA) control obtained using GenomePlex^®^ Whole Genome Amplification Kit (Sigma-Aldrich, Steinheim, Germany).

### Histone mark H3K27ac ChIP-seq (Chromatin immunoprecipitation-sequencing)

Cross-linking of cHL cells (7 cHL cell lines) and 3 germinal center B-cell pools (GCBs), (~10*10^6^ cells per cell line, cHL and GCB respectively) was performed by incubating the cells in 1% formaldehyde for 8 minutes in RT. Thereafter, genomic DNA was sonicated using Covaris™ E220 device in order to obtain 50–500 bp length DNA fragments. Chromatin preparation and histone ChIP were performed according to the BLUEPRINT protocols (https://www.blueprint-epigenome.eu/index.cfm?p=7BF8A4B6-F4FE-861A-2AD57A08D63D0B58) using the anti-H3K27ac (Diagenode C15410196, lot no. A1723-0041D) antibody. The library for high-throughput sequencing was prepared using Kapa Hyper Prep Kit (Kapa Biosystems, Roche, Basel, Switzerland) according to the BLUEPRINT protocol. Briefly, end repair and A-Tailing was followed by adapter ligation. Library amplification was performed in the following conditions 98°C for 45 s x1; 98°C for 15 s, 60°C for 30 s, 72°C for 30 s x 12; 72°C for 1 min; 12°C ∞. PCR products were cleaned using AMPure XP beads followed by size selection of DNA fragments (300 bp) with E-Gel™ SizeSelect™ (Invitrogen, Waltham, Massachusetts, USA).

High-throughput sequencing was carried out using the HiSeq2500 (Illumina) sequencer [36]. Raw reads (fastq) were mapped to the GRCh38 reference genome using BWA aligner (aln, samse) [37]. Obtained bam files were sorted (Samtools) and peaks were called using MACS2 [38,39]. DESeq2 R package was used in order to determine the active and inactive chromatin regions in cHL compared to GCB controls [40].

### *In silico* functional enrichment analysis of novel miRNA target genes

In order to identify potential gene targets for the novel experimentally confirmed miRNAs two freely available tools were used: miRDB [41] and miRanda [42] allowing for custom target prediction. The complete set of human genes (3’UTRs) was used as the reference. This analyses resulted in two cohorts of potential targets for each of the novel miRNAs. We further selected potential target genes according to the following criteria:

- total score > 280 for miRanda and > 80 for miRDB,
- only genes present in both cohorts for each of the analysed novel miRNA respectively.

These gene sets were used as the input for Gene Ontology (GO) analyses performed using STRING [43] and GO Consortium database [44,45]. Finally, we selected the common set of biological processes returned from both tools.

### Further validation of novel miRNA candidates

For additional validation of novel miRNA candidates, cDNA templates and RT-qPCR products were obtained using Custom TaqMan™ Small RNA Assay (Thermo Fisher Scientific) with the same custom miRNA probes as descripted above (**S1 Table**). The RT-qPCR products were visualized on 1.8% agarose gel with SimplySafe™ (EURx) under UV light (BioDoc‐it Imaging System, UVP, USA) and purified using DNA Clean & Concentrator–5 Kit (Zymo Researh, Germany). PCR products were subjected to A-tailing procedure (30 minutes, 70 ⁰C) followed by ligation reaction with pGEM ®-T Easy Vector (Promega; 16 hours in 4 ⁰C). Obtained constructs were used for transformation of JM109 *E*.*coli* competent cells (Promega). Lastly, colony PCR was performed using M13 primers (**S5 Table**) according to the following protocol: 95°C for 2 min × 1; (95°C for 30 s, 55°C for 30 s, 72°C for 30 s) × 30, 72°C for 5 min × 1. Obtained PCR products were then visualised, purified and sequenced (Sanger) as described previously [25].

## Results

### Identification of novel miRNAs expressed in cHL cell lines

The analysis of the miRNA-seq data from our previous study [9] resulted in the identification of 1303 miRNA-like sequences with no miRBase annotation (release 22.1) [27]. The vast majority of these sequences showed very low levels of expression, therefore we excluded all miRNAs expressed below 20 CPM. This filtering resulted in 19 sequences which were manually curated to discard potential known molecules other than miRNAs as well as sequencing artefacts. Moreover, using published data of the ICGC MMML-Seq [28] we aimed to identify miRNAs potentially involved in cHL pathogenesis. We rejected candidates observed more frequently than in 2% of the NHL cohort (n = 56). Altogether, 6 novel miRNA candidates were identified and further validated experimentally.

**Fig 1:**
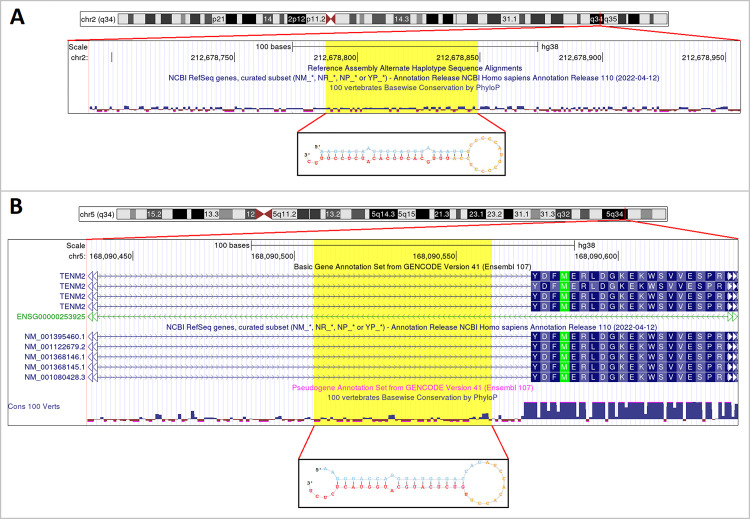
Representation of two confirmed novel miRNAs. Genomic position (GRCh38) and structure of confirmed novel miRNAs. **A:** 2_nv_chr2_212678788; **B:** 3_nv_chr5_168090507. Mature miRNA sequences are shown in red.

**Fig 2:**
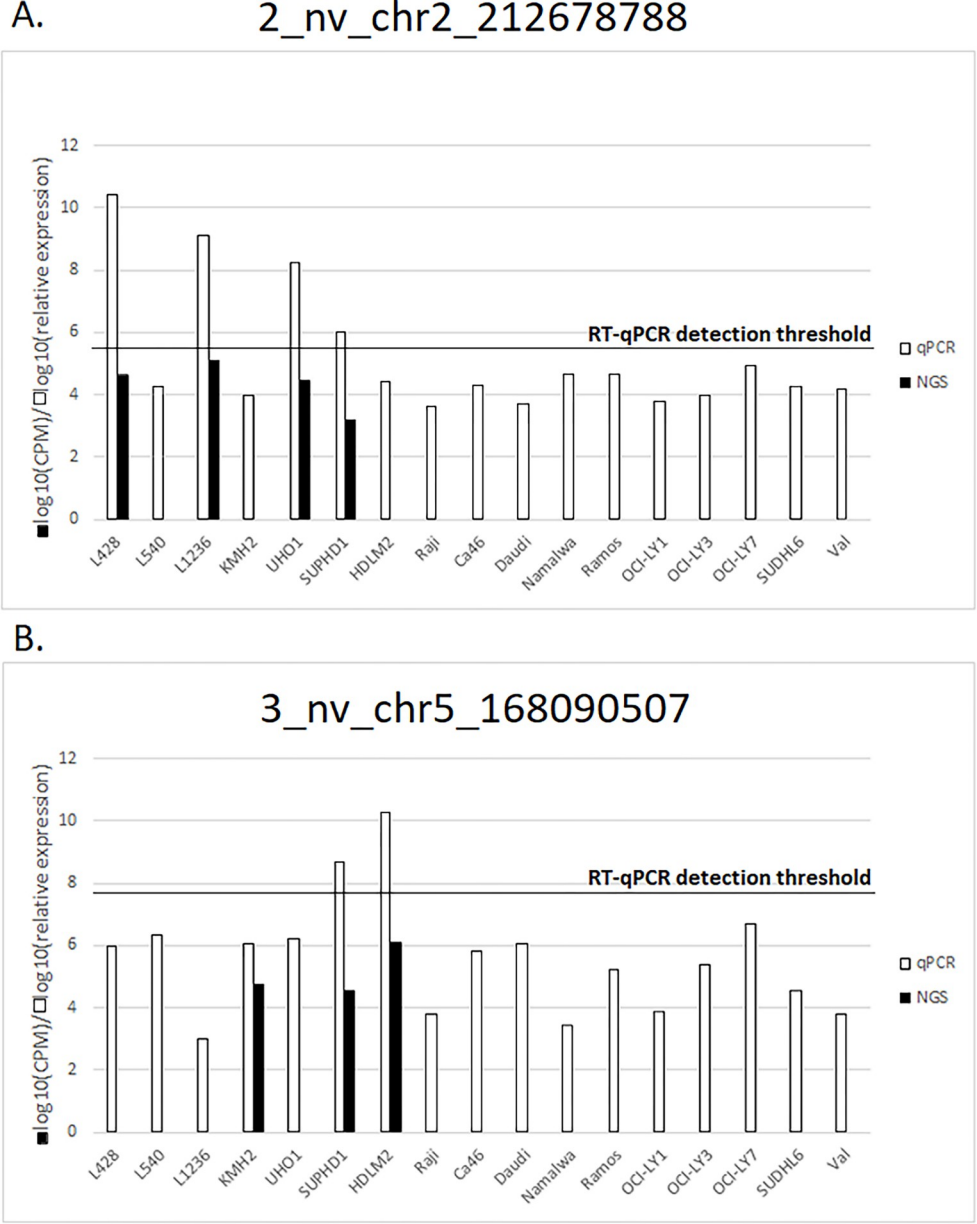
The comparison of log10 normalized relative expression of validated miRNAs. Black bars represent NGS experiment (CPM) and white bars the RT-qPCR relative expression. The RT-qPCR detection thresholds were obtained by adding the standard deviation of the RT-qPCR results to the mean relative expression level for each miRNA, respectively.

All six selected miRNAs were tested using TaqMan^TM^ probes (RT-qPCR). Two out of the six novel miRNA candidates were confirmed to be expressed in the cHL cell lines (**Fig 1; Table 1**). The novel miRNA (2_nv_chr2_212678788) was confirmed to be expressed by RT-qPCR in 4/7 cHL cell lines (SUP-HD1, U-HO1, L-428, L-1236) and absent in the remaining cHL and NHL cell lines in agreement with the RNA-seq data (**Fig 2A**).

**Table 1:** Characterization of novel miRNA candidates. Expression of the bolded candidates was confirmed using RT-qPCR with TaqMan^TM^ probes.

Chr	Start (GRCh38)	Stop (GRCh38)	Novel miRNA candidate ID	Expressed in cHL cell lines (NGS)	Mean CPM	Expressed in non-HL cases	Validation in cHL cell lines (RT-qPCR)
**chr2**	**212678788**	**212678849**	**2_nv_chr2_212678788**	**L-428, L-1236, U-HO1,** **SUP-HD1**	**23**	**0/56 (0%)**	**Confirmed in 4/4 cell lines**
**chr5**	**168090507**	**168090561**	**3_nv_chr5_168090507**	**KM-H2,** **SUP-HD1,** **HDLM-2**	**40**	**1/56 (1.7%)**	**Confirmed in 2/3 cell lines**
chr6	1475207	1475275	1_nv_chr6_1475207	L-428, U-HO1, HDLM-2	74	0/56 (0%)	Not confirmed
chr6	149118294	149118354	6_nv_chr6_149118294	L-428, L540, HDLM-2	35	0/56 (0%)	Not confirmed
chr7	9978163	9978226	7_nv_chr7_9978163	L-428, U-HO1, SUP-HD1	22	0/56 (0%)	Not confirmed
chr19	6613612	6613674	4_nv_chr19_6613612	L-428, L-540, SUP-HD1	27	0/56 (0%)	Not confirmed

The second novel molecule (3_nv_chr5_168090507) was confirmed to be expressed by RT-qPCR in 2 of 3 cHL cell lines (SUP-HD1, HDLM-2 but not KM-H2) indicated initially by the NGS experiment (**Fig 2B**). In summary, these analyses allowed the identification of two novel miRNAs recurrently expressed in cHL cell lines.

### Validation of 2_nv_chr2_212678788 and 3_nv_chr5_168090507 miRNA expression in cHL cell lines

We next performed an additional validation step of the two unannotated miRNAs (nv_chr2_212678788 and 3_nv_chr5_168090507). We used an alternative reverse transcription kit which allows individual amplification of the miRNAs of interest followed by qPCR with the same custom probes as used previously.

Importantly, we observed expression of particular unannotated miRNAs in the same cHL cell lines as indicated by both NGS and previous RT-qPCR experiments (alternative approach). Lastly, we cloned the obtained PCR products and performed Sanger sequencing. It shoved the presence of particular unannotated miRNA sequences (mature miRNA molecules of nv_chr2_212678788 and 3_nv_chr5_168090507) in the respective cHL cell lines (**S6 Table**).

### 2_nv_chr2_212678788 and 3_nv_chr5_168090507 precursors undergo physiological processing yielding mature miRNAs

**Fig 3:**
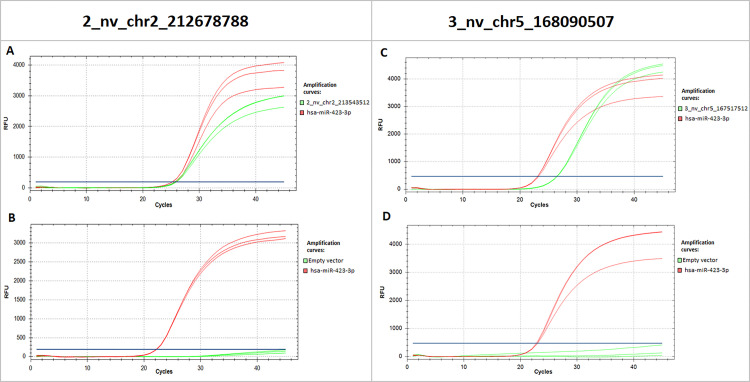
Expression of novel miRNAs in transfected HEK-293 cell line. RT-qPCR amplification curves showing expression of the mature sequences of novel miRNAs in the HEK-293 cell line transfected with the constructs containing the novel miRNA genes (upper panel) and empty vector (lower panel) respectively. Green curves represent expression of mature novel miRNAs **A:** of 2_nv_chr2_212678788 and **B:** of 3_nv_chr5_168090507. Red curves represent the expression of the reference hsa-miR-423-3p.

To analyze if 2_nv_chr2_212678788 and 3_nv_chr5_168090507 undergo canonical miRNA biogenesis we cloned the PCR products containing the entire miRNA precursor sequences into the pCDH-CMV-MCS-EF1α-Green Puro cloning vector and used these constructs to transfect the HEK-293 cell line. We recurrently observed the expression of both novel miRNAs after transfection with the miRNA constructs, whereas no expression of the novel miRNAs was observed in samples transfected with the empty vector (**Fig 3**). In summary, these results demonstrate that 2_nv_chr2_212678788 and 3_nv_chr5_168090507 precursors are processed by the cell into mature miRNAs identified using the TaqMan^TM^ assays.

### DNA hypomethylation and chromatin activation drives expression of the novel miRNA 3_nv_chr5_168090507

**Fig 4:**
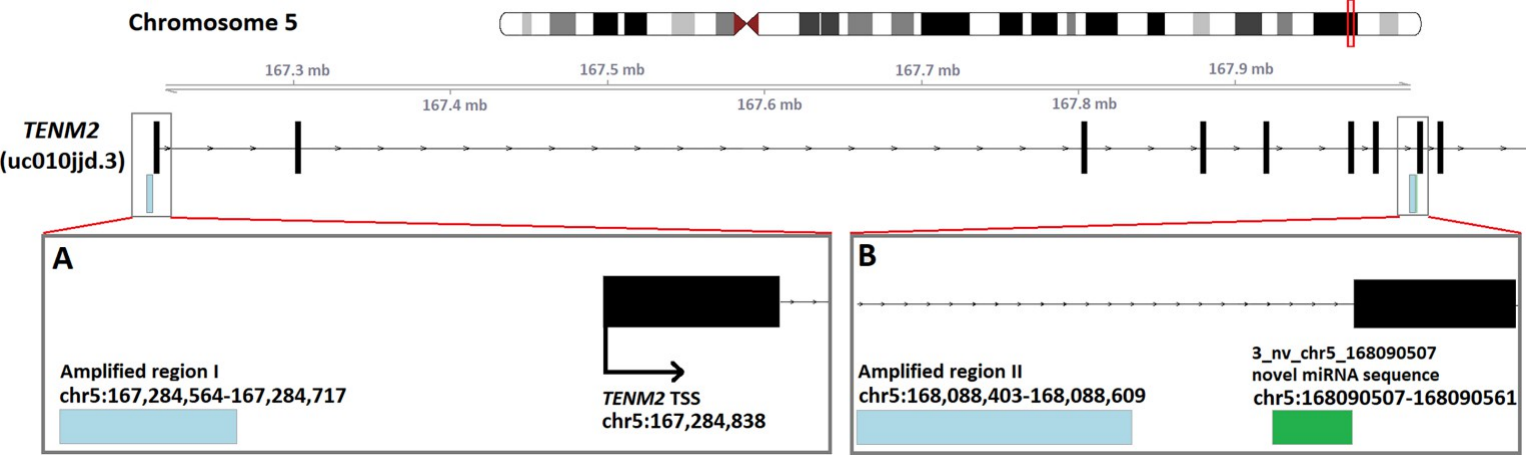
Visualisation of the genomic positions (GRCh38) of bisulfite pyrosequencing assays used for DNA methylation analysis of the novel 3_nv_chr5_168090507 miRNA. Two regions were examined (blue). Region (I) 166 bp upstream of TSS of *TENM2* **(A)** and region (II) adjacent (~2 kb upstream) to novel miRNA gene (*TENM2* intron) **(B)**.

As HRS cells are characterised by global epigenetic reprogramming which includes alteration of DNA methylation of promoter regions and loss of epigenetic control of LTRs. We hypothesized that the expression of the two novel miRNAs 3_nv_chr5_168090507 and 2_nv_chr2_212678788 is triggered by specific DNA hypomethylation in the analyzed cell lines. In order to test this hypothesis three pyrosequencing assays were designed to analyze for potential hypomethylation in the respective regions. DNA methylation levels in cHL cell lines were compared to NHL cell lines that do not express these novel miRNAs. Because of the intronic localization of the 3_nv_chr5_168090507 miRNA gene, it can be transcribed canonically, as a separate miRNA gene, or non-canonically together with the large *TENM2* transcript (uc010jjd.3 –largest gene transcript with the highest level of review) as a mirtron [46]. Therefore, for this novel miRNA, two pyrosequencing assays were designed to test both possibilities. The first in the vicinity of the miRNA (~2 kb upstream, putative miRNA gene, *TENM2* intronic sequence) and the second region 166 bp upstream of the *TENM2* gene TSS (**Fig 4**). The second analyzed miRNA (2_nv_chr2_212678788) is located in a gene desert, in a THE1C LTR, therefore only the region within the LTR and adjacent to the miRNA was tested (54 bp upstream).

**Fig 5:**
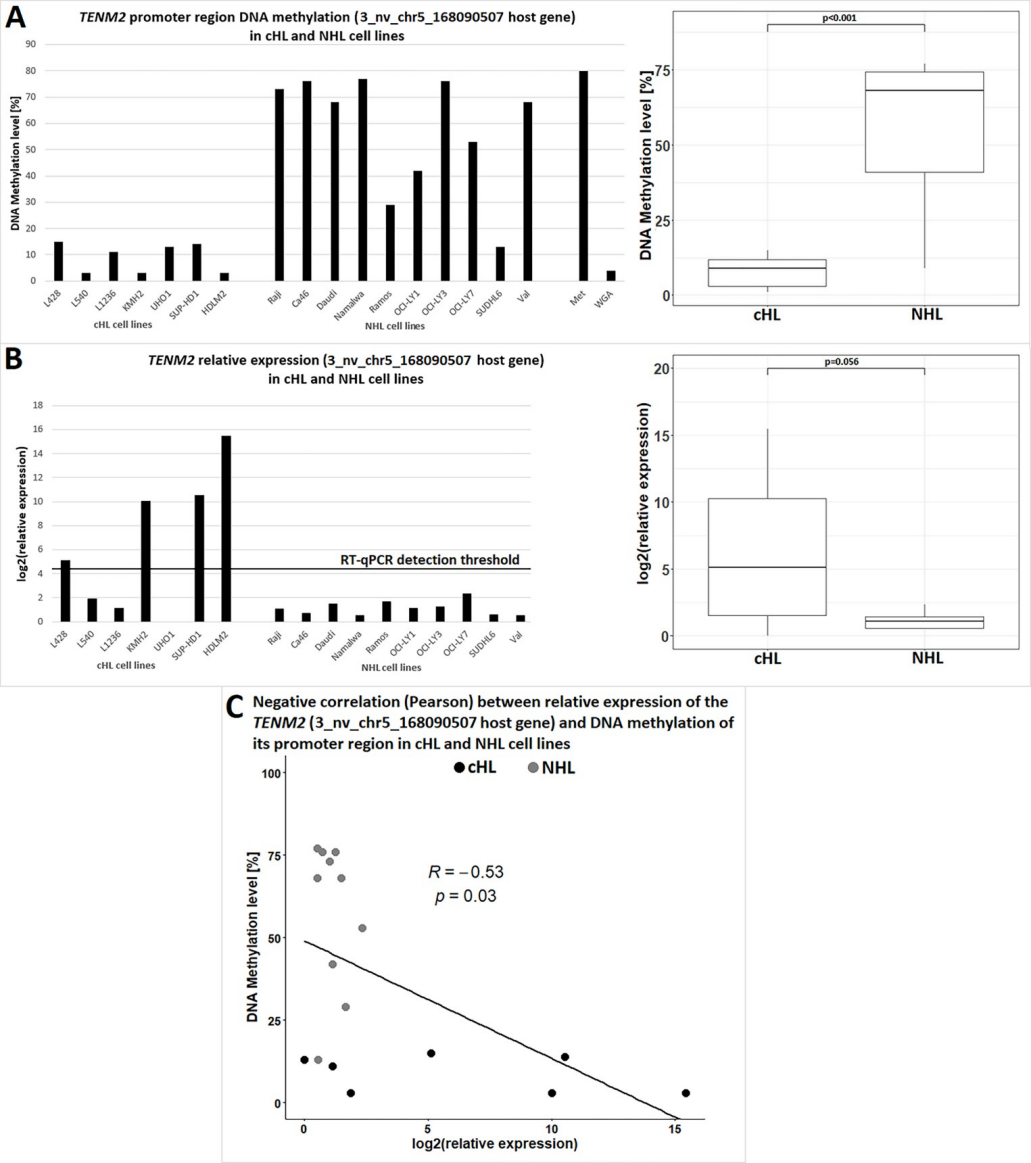
Comparison of DNA methylation level of the *TENM2* promoter region with relative expression of this gene. Bars represent methylation of one CG dinucleotide analysed by the bisulfite pyrosequencing assay **(A)** and *TENM2* relative expression **(B)**. Statistically significant negative correlation between *TENM2* relative expression and DNA methylation in cHL (black dots) and NHL cell lines (grey dots) **(C)**.

The mean methylation level in the vicinity of the 3_nv_chr5_168090507 miRNA gene (assay II; 7 CG dinucleotides) was 79% (range: 1–100; sd = 24) in the cHL cell lines and 82% (range: 2–97%; sd = 17) in the NHL cell lines showing no significant difference in DNA methylation level between the studied lymphoma types. Similarly, no differences were observed for the LTR located 2_nv_chr2_212678788 miRNA for which the mean methylation level (3 CG dinucleotides) was 76% (range: 5–99%; sd = 28) in the cHL cell lines and 75% (range: 24–99%; sd = 19) in the NHL cell lines. This finding suggests that activation 2_nv_chr2_212678788 miRNA is not a direct effect of the respective LTR hypomethylation. However, we observed significant hypomethylation (p<0.05) of the *TENM2* promoter region (host gene of 3_nv_chr5_168090507 miRNA) using assay I; 1 CG dinucleotide (**Fig 5A and 5B**) in cHL cell lines (mean methylation level 9%: range: 3–15%; sd = 5) compared to NHL cell lines (mean methylation level: 57.5% range: 13–77%; sd = 21). Summarizing, DNA hypomethylation of the *TENM2* promoter region is a potential mechanism responsible for 3_nv_chr5_168090507 transcriptional activation in cHL.

Moreover, to test if hypomethylation observed in the *TENM2* promoter region indeed results in elevated expression of the gene (3_nv_chr5_168090507 primary transcript) we performed RT-qPCR to quantify *TENM2* expression. It indicated a negative correlation (R<-0.5, p<0.05) (**Fig 5C**) between the level of expression and DNA methylation of *TENM2* promoter region. These data suggest that *TENM2* is epigenetically regulated in cHL and NHL cell lines. The expression of miRNA 3_nv_chr5_168090507 in the KM-H2, SUP-HD-1, and HDLM-2 cell lines, which also showed the highest *TENM2* expression, renders it likely that this novel miRNA is transcribed together with the *TENM2* host-gene.

**Fig 6:**
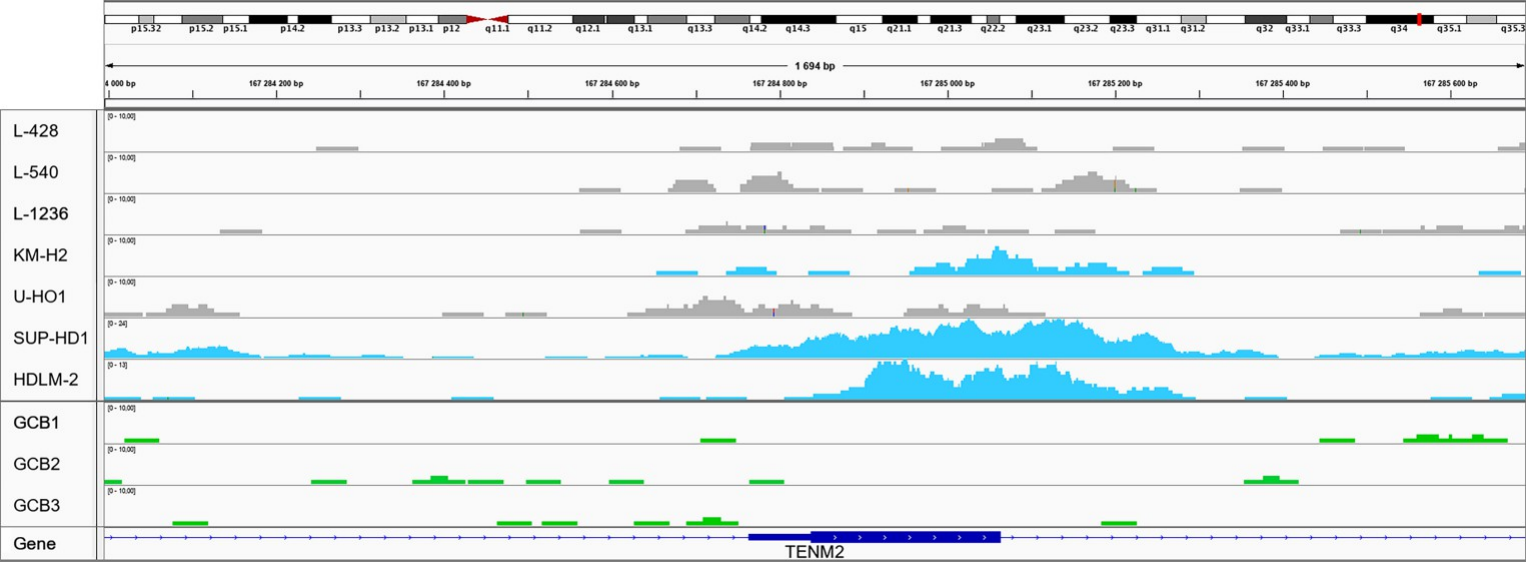
*TENM2* ChIP-seq results in cHL cell lines and GCBs. The visualisation (IGV) [47] of ChIP-seq (H3K27ac) experiment performed on 7 cHL cell lines and 3 GCB cell pools (left panel). The 27^th^ lysine acetylation for *TENM2* promoter region shows higher chromatin activity in KM-H2, SUP-HD1 and HDLM-2 cHL cell lines (blue peaks). Grey peaks represent cHL cell lines with less activated chromatin, whereas chromatin is closed in the GCB controls (green peaks).

In order to further confirm this mechanism we studied ChIP-seq data (H3K27ac) of the 7 cHL cell lines, 3 GCB cell pools (controls) and 5 NHL cell lines (controls, external data). We focused on the *TENM2* promoter region to look for changes in the chromatin activity. The experiment showed open chromatin regions near proximal *TENM2* TSS the cHL cell lines compared to fully closed chromatin in the GCB cell controls (**Fig 6**) and the studied NHL cell lines: BL2, DG-75, KARPAS-422, SU-DHL-5, Z-138 (**S1 Fig**). The highest chromatin activity was observed in KM-H2, SUP-HD-1 and HDLM-2 cell lines (**Fig 6**) what translates into elevated relative expression of *TENM2* and the novel miRNA in these cHL cell lines **(Fig 5B)**.

### Expression of novel miRNA 3_nv_chr5_168090507 in primary microdissected HRS cells

In order to test if overexpression of the novel 3_nv_chr5_168090507 miRNA is only due to the deregulation of cHL cell lines or is also found in primary cHL cases, we next analysed its expression in 10 primary microdissected HRS cell pools. No expression of the novel miRNA 3_nv_chr5_168090507 was observed in the HRS cells (**S2 Fig**), what in part may be caused by the low performance of the TaqMan probe in the microdissected samples. Therefore, the novel miRNA 3_nv_chr5_168090507 is expressed only in the analysed cHL cell lines or its expression level in the microdissected HRS cells is beyond the detection level of the used technique.

### The novel 2_nv_chr2_212678788 is localized in long tandem repeat region

**Fig 7:**
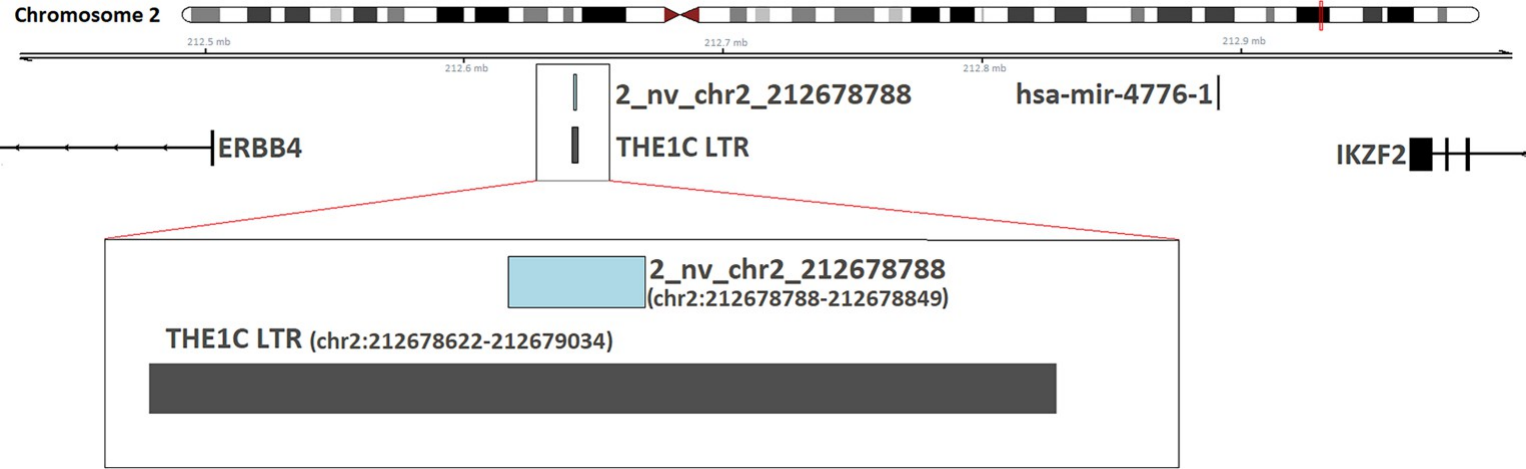
Visualisation of 2_nv_chr2_212678788 novel miRNA genomic position. Genomic position (GRCh38) of novel miRNA 2_nv_chr2_212678788 sequence located in THE1C LTR.

It has recently shown, that aberrant activation of more than 1800 LTRs in cHL participates in global transcriptional deregulation of protein coding genes in this lymphoma [48]. Moreover, we have previously shown that this phenomenon leads to a cHL specific expression of the oncogene *CSF1R* that is crucial for the survival of HRS cells [49]. In light of these findings we found it particularly interesting that the novel miRNA 2_nv_chr2_212678788 is localized within a LTR THE1C from ERVL-MaLR family (**Fig 7**) that triggers the tempting speculation that its aberrant expression may be driven by the LTR’s transcriptional re-activation [49]. We therefore mined the RACE-seq data published by Edginton-White et al. but found no transcriptional activity reported within this region [48]. This finding is consistent with our ChIP-seq data (H3K27ac) which showed closed chromatin in this region in all 7 cHL cell lines as well as 3 GCB cell pools used as controls. In conclusion, the mechanism which drives the observed expression of miRNA 2_nv_chr2_212678788 is probably not related to aberrant activation of LTRs in cHL.

### The novel 2_nv_chr2_212678788 miRNA is involved in various regulatory processes

Based on the criteria outlaid in the methods section, custom miRNA target prediction for the novel miRNAs resulted in 45 genes (miRDB (http://mirdb.org/), miRanda (http://cbio.mskcc.org/miRNA2003/miranda.html)) for 2_nv_chr2_212678788 and 9 genes for 3_nv_chr5_168090507 indicated by both tools. The GO analysis performed using obtained cohorts of genes, resulted in 13 biological processes for 2_nv_chr2_212678788 (indicated independently by both tools, STRING, GO Consortium; FDR < 0.05) (**S7 Table**) and no biological process for 3_nv_chr5_168090507. For 2_nv_chr2_212678788 these included several processes responsible for regulation of gene expression, metabolic processes and circadian regulation. Although, these processes are generally related to malignant transformation, no ontologies typically associated with cHL pathogenesis were identified.

## Discussion

MicroRNAs are important factors involved in post-transcriptional regulation of gene expression. These molecules were also shown to play a substantial role in various processes related to lymphocyte proliferation and differentiation [50].

We and other groups have recently performed high throughput experiments to characterize the miRnome of cHL cell lines [9,51,52]. In the present study we reanalysed this dataset and identified 6 promising novel miRNA candidates expressed exclusively in cHL cell lines. Two of them were successfully confirmed using RT-qPCR with custom TaqMan^TM^ probes and downstream analyses. However, both validated miRNAs as well as the other four candidates were characterized by low relative expression (CPM below 100). It may potentially explain why only two of the six novel miRNAs were confirmed experimentally. Alternatively, despite of accurate filtering of false positive results, these four unconfirmed miRNAs may be in fact low expressed artifacts wrongly annotated as miRNAs.

By cloning and transfecting the precursor sequences of the two novel miRNAs (2_nv_chr2_212678788 and 3_nv_chr5_168090507) to the HEK-293 cell line and downstream detection of the mature sequences we demonstrated that both miRNAs are biologically processed by the cell. Therefore, these two yet unannotated miRNAs are potentially functional. However, their low expression level leaves doubts on their physiological relevance in cHL. In addition, the qRT-PCR experiments on microdissected HRS cells did not confirm the presence of these miRNAs in the primary cells. This may be on one hand due to technical reasons investigating a small number of the analyzed, pooled, microdissected cells (1000) which in combination with the low expression of these miRNAs may be below the detection level of the technique. On the other hand, this may also indicate that their low expression is a side effect of the global transcriptional reprogramming of the studied cHL cell lines or it may be indicative for therapy-refractory disease. In line with this assumption we observed DNA hypomethylation of *TENM2* gene (region <200 bp upstream of TSS) harboring the 3_nv_chr5_168090507 miRNA, in cHL cell lines compared to NHL cell lines. Moreover, we noticed the expression of *TENM2* (uc010jjd.3 transcript) exclusively in the KM-H2, SUP-HD-1 and HDLM-2 cell lines that correlates to elevated chromatin activity (H3K27ac ChIP-seq) around the *TENM2* TSS in these cell lines. Thus, our results indicate that DNA hypomethylation is the mechanism responsible for transcriptional activation of both, the *TENM2* gene itself and the 3_nv_chr5_168090507 miRNA in cHL cell lines. Noteworthy, the *TENM2* gene was not reported to be associated with cHL pathogenesis itself.

In contrast to 3_nv_chr5_168090507 miRNA we did not identify the mechanism behind the activation of miRNA 2_nv_chr2_212678788.

Finally, we performed *in silico* GO analysis using the 45 (2_nv_chr2_212678788) and 9 (3_nv_chr5_168090507) target genes of these miRNAs as input. The analysis indicated an enrichment by terms related to metabolism and regulation of gene expression. How far these processes reflect malignant transformation in cHL remains to be studied.

## Conclusions

In this study we discovered two unannotated miRNAs expressed exclusively in cHL cell lines. Using a functional approach, we proved that the molecules undergo a typical processing for miRNAs resulting in the expression of mature miRNA sequences. Moreover, we identified DNA hypomethylation and chromatin activity as mechanism for the transcriptional activation of 3_nv_chr5_168090507 miRNA in cHL cell lines.

## Supporting information

S1 Fig *TENM2* chromatin activity profile. The visualisation (UCSC) of data from BLUEPRINT project (https://www.blueprint-epigenome.eu/index.cfm?p=3D6C68FA-3048-9110-625D850E3E055A84) regarding chromatin activity of *TENM2* promoter region (vertical blue bar showed on upper panel). The ChIP-seq (H3K27ac) experiment was performed on 5 NHL cell lines. The horizontal bars near particular cell line name represent chromatin state. Black colour of the bar indicates inactive heterochromatin region, whereas other colours are showing active states of chromatin. The 27^th^ lysine acetylation for *TENM2* promoter region shows fully closed chromatin regions in all NHL cell lines.
(TIF)

S2 Fig Novel miRNA expression in HRS cells. Expression of 3_nv_chr5_168090507 novel miRNA in 10 primary microdissected HRS cell pools with corresponding membrane negative controls. (cHL cell lines (green) and GC B cells (red) are shown as positive and negative controls, respectively.
(TIF)

S1 Table TaqMan^TM^ probe sequences. Sequences of custom TaqMan^TM^ probes used for validation of novel miRNA candidates and sequences of two known miRNAs used as the reference.
(DOCX)

S2 Table *TENM2* expression primers. Primer sequences used for TENM2 mRNA expression analysis.
(DOCX)

S3 Table Amplified sequences used in functional analysis. Genomic locations of amplified regions (GRCh38) and primer sequences used for amplification of two novel miRNA genes. The amplicons were used in further functional analysis. Detailed information about the amplified regions: whole amplified sequence, miRNA precursor sequence (green), mature miRNA sequence (highlighted in yellow) is presented below the table.
(DOCX)

S4 Table Bisulfite DNA pyrosequencing primers. Genomic locations of amplified regions (GRCh38) and primer sequences used for bisulfite DNA pyrosequencing (DNA methylation analysis) of three regions corresponding to the two novel miRNA candidates.
(DOCX)

S5 Table pGEM ®-T Easy Vector primer sequences. pGEM ®-T Easy Vector primer sequences used for amplification and Sanger sequencing of nv_chr2_212678788 and 3_nv_chr5_168090507 novel miRNA sequences.
(DOCX)

S6 Table Results of colony PCR sequencing. Sequencing of colony PCR products (pGEM-T Easy Vector with cloned novel miRNA insert obtained by qPCR). The qPCR reaction was performed using Custom TaqMan™ Small RNA assays. Last column shows the sequenicng chromatograms with respective novel miRNA sequence highlighted in blue.
(XLSX)

S7 Table Functional enrichment analysis results. Functional enrichments found in the group of 45 genes that are the putative targets of the novel 2_nv_chr2_212678788 miRNA expressed in cHL cell lines (STRING, GO Consortium).
(DOCX)
