# Supplementary figures and images for: Identification of two unannotated miRNAs in classic Hodgkin lymphoma cell lines
Source: PLoS One. 2023 Mar 24;18(3):e0283186. doi: 10.1371/journal.pone.0283186 (PMC10038261; doi:10.1371/journal.pone.0283186)

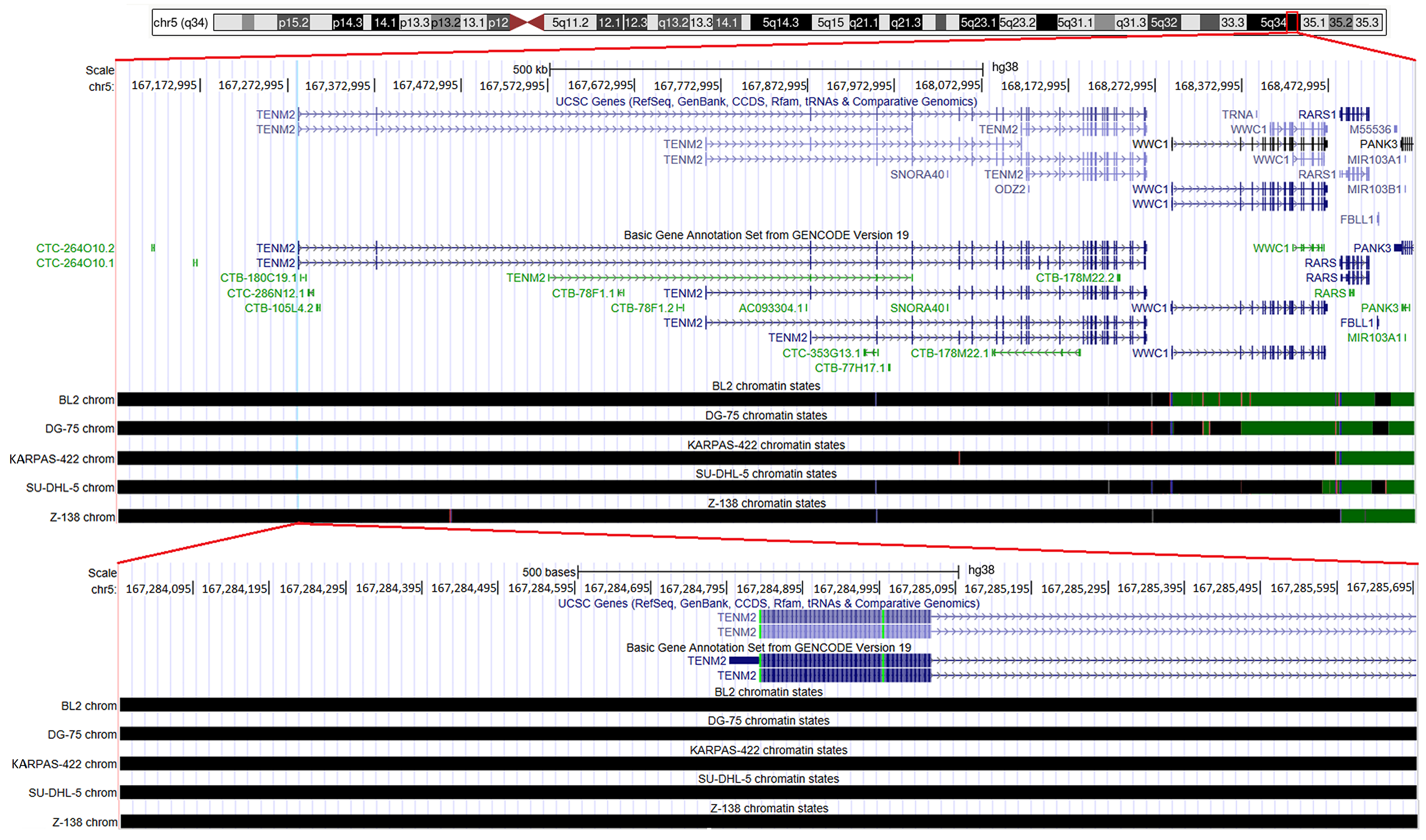

Supplement: S1 Fig — The visualisation (UCSC) of data from BLUEPRINT project (https://www.blueprint-epigenome.eu/index.cfm?p=3D6C68FA-3048-9110-625D850E3E055A84) regarding chromatin activity of TENM2 promoter region (vertical blue bar showed on upper panel). The ChIP-seq (H3K27ac) experiment was performed on 5 NHL cell lines. The horizontal bars near particular cell line name represent chromatin state. Black colour of the bar indicates inactive heterochromatin region, whereas other colours are showing active states of chromatin. The 27th lysine acetylation for TENM2 promoter region shows fully closed chromatin regions in all NHL cell lines. (TIF) [file pone.0283186.s001.tif]

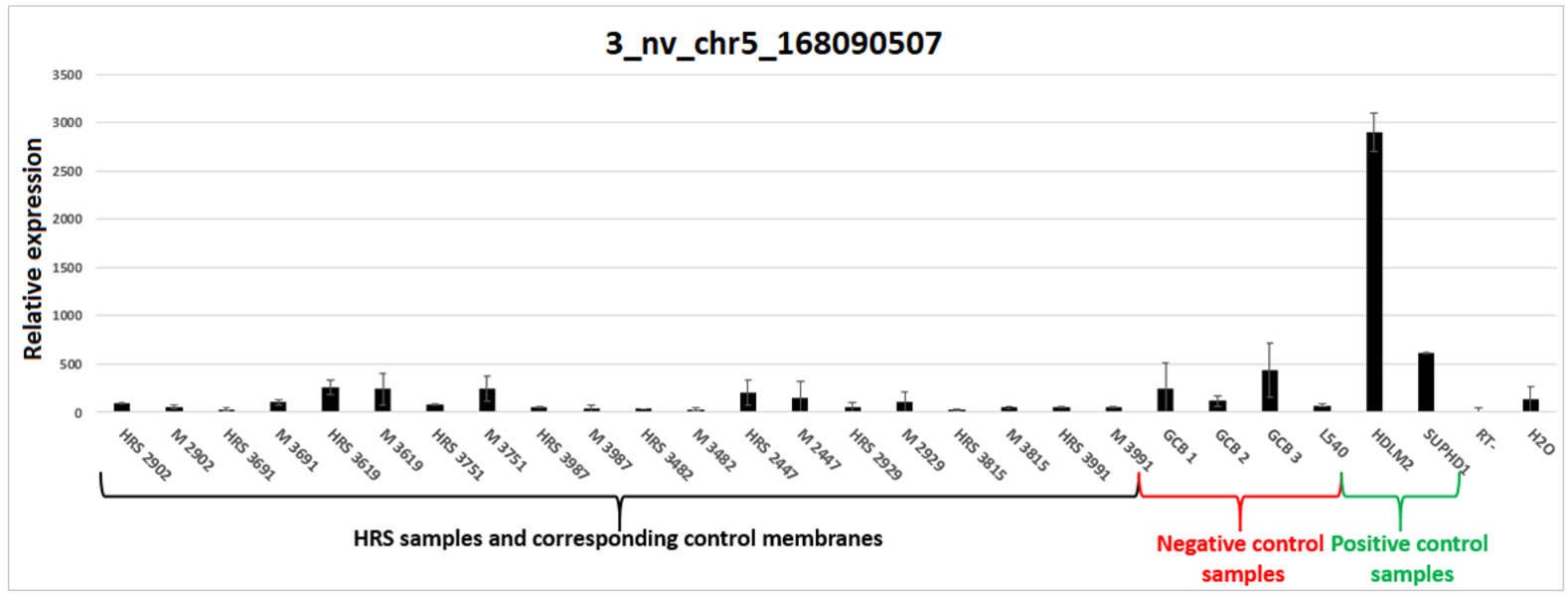

Supplement: S2 Fig — Expression of 3_nv_chr5_168090507 novel miRNA in 10 primary microdissected HRS cell pools with corresponding membrane negative controls. (cHL cell lines (green) and GC B cells (red) are shown as positive and negative controls, respectively. (TIF) [file pone.0283186.s002.tif]
