# Supplementary material for: Identification of two unannotated miRNAs in classic Hodgkin lymphoma cell lines
Source: PLoS One. 2023 Mar 24;18(3):e0283186. doi: 10.1371/journal.pone.0283186 (PMC10038261; doi:10.1371/journal.pone.0283186)
Supplement: S1 Table — Sequences of custom TaqManTM probes used for validation of novel miRNA candidates and sequences of two known miRNAs used as the reference. (DOCX) [file pone.0283186.s003.docx]

**Table S1. TaqMan^TM^ probe sequences.** Sequences of custom TaqMan^TM^ probes used for validation of novel miRNA candidates and sequences of two known miRNAs used as the reference.

| **Novel miRNA candidate ID / reference miRNA name** | **TaqMan™ probe sequence 5’ -> 3’** | **Consensus mature miRNA sequence 5’ -> 3’** |
| --- | --- | --- |
| 2_nv_chr2_212678788 | uuugcacugcacaucuccuuu | uuugcacugcacaucuccuu |
| 3_nv_chr5_168090507 | ugucucaugcaugguacucucu | ugucucaugcaugguacucucu |
| 1_nv_chr6_1475207 | cauggcauacuacugagcagu | cauggcauacuacugagcagu |
| 6_nv_chr6_149118294 | uuggggcagugagggucugugu | uuggggcagugagggucugugu |
| 7_nv_chr7_9978163 | auuugcuucugugauaacauga | auuugcuucugugauaacauga |
| 4_nv_chr19_6613612 | uuacauugggauuagagacaag | uacauugggauuagagacaaga |
| hsa-miR-191-5p (reference, validation of NGS experiment) | caacggaaucccaaaagcagcug | caacggaaucccaaaagcagcug |
| hsa-miR-361-5p (reference,  validation of NGS experiment) | uuaucagaaucuccagggguac | uuaucagaaucuccagggguac |
| hsa-miR-423-3p (reference, validation of novel miRNAs biogenesis) | agcucggucugaggccccucagu | agcucggucugaggccccucagu |
