# Supplementary material for: Identification of two unannotated miRNAs in classic Hodgkin lymphoma cell lines
Source: PLoS One. 2023 Mar 24;18(3):e0283186. doi: 10.1371/journal.pone.0283186 (PMC10038261; doi:10.1371/journal.pone.0283186)
Supplement: S2 Table — Primer sequences used for TENM2 mRNA expression analysis. (DOCX) [file pone.0283186.s004.docx]

**Table S2. *TENM2* expression primers.** Primer sequences used for *TENM2* mRNA expression analysis.

| **Gene** | **Primer F 5' -> 3'** | **Primer R 5' -> 3'** |
| --- | --- | --- |
| *TENM2* | TCCCTTAGCACAGGGTCTGA | GAGCTAGGCAGCTGAGCAGA |
| *ACTB* | CACCACACCTTCTACAATG | TAGCACAGCCTGGATAG |
| *GAPDH* | GTCGGAGTCAACGGATT | CCTGGAAGATGGTGATGG |
