# Supplementary material for: Identification of two unannotated miRNAs in classic Hodgkin lymphoma cell lines
Source: PLoS One. 2023 Mar 24;18(3):e0283186. doi: 10.1371/journal.pone.0283186 (PMC10038261; doi:10.1371/journal.pone.0283186)
Supplement: S3 Table — Genomic locations of amplified regions (GRCh38) and primer sequences used for amplification of two novel miRNA genes. The amplicons were used in further functional analysis. Detailed information about the amplified regions: whole amplified sequence, miRNA precursor sequence (green), mature miRNA sequence (highlighted in yellow) is presented below the table. (DOCX) [file pone.0283186.s005.docx]

**Table S3. Amplified sequences used in functional analysis.** Genomic locations of amplified regions (GRCh38) and primer sequences used for amplification of two novel miRNA genes. The amplicons were used in further functional analysis. Detailed information about the amplified regions: whole amplified sequence, miRNA precursor sequence (green), mature miRNA sequence (highlighted in yellow) is presented below the table.

| **Novel miRNA gene** | **Primer F 5' -> 3'** | **Primer R 5' -> 3'** | **Genomic location of amplified region (GRCh38)** | **Genomic location of novel miRNA precursor sequence (GRCh38)** | **Genomic location of novel miRNA mature sequence (GRCh38)** |
| --- | --- | --- | --- | --- | --- |
| 2_nv_chr2_212678788 | TGCAAAGTTTCATCCTTCCAG | GTGAAATCTGTGAACTGAAAATCA | chr2:212678556-212679130 | chr2:212678788-212678849 | chr2:212678791-212678810 |
| 3_nv_chr5_168090507 | CCATCATTGTAGAAGGCCAGA | ACAAGAGATTGTGGCCTAGAGT | chr5:168090330-168090728 | chr5:168090507-168090561 | chr5:168090539-168090560 |

| >chr2:212678556-212679130; 2_nv_chr2_212678788; (GRCh38)  TGCAAAGTTTCATCCTTCCAGATTCCCTACATCTAACTTCCTTTTTACTGTAAATAACTCGGGTGGTGTAATAGTCCGTTCTTATGCTGCTATAAAACAACTTCCCAAGACTGGGTAATTCATAAAGAAAAGAAGTTCAGTTGACTCGCAGTTCTGCAGGGTTAGGGAAGCCTCAGGAAACTTACAATCATGGTGGAAAGGGAAGCAAACACATCCTTCTTCACGTGGCAACAGAAAGGAGATGTGCAGTGCAAATGGAGGGGCATGGGGGCCATTTTCACTGCACTTCTCCTTCCTGTTGCCATGTATAAAACCATCAGACCTCATGAGAACTCACTTACTGTCATGAGAACAGCATGGGGGAACCGCTGCCATGATCTGTCGCCTCCCGTGAGGTTCCTCCCCCAACACATGGGGATTACAATTGGGATTACAATTCAAGATGAGATTTGGGTGGGGATACAGAGCCAAACCATATCTTCCACTTTCCTGACCAAATTTAACTTATACATGAAGCAAATCCTCAAACATTCAAACAACAATTCAGATAATGATTTTCAGTTCACAGATTTCAC |  |
| --- | --- |

|  | >chr5:168090330-168090728; 3_nv_chr5_168090507; (GRCh38)  ACAAGAGATTGTGGCCTAGAGTAATAAAAGTATTAATAGTAGCAATAATTATAAAGCATAATAAATAATGACTGTTATTATTGTTACACATTTTGCGGCTCTTGTTTTCCTGGATAAGTTCATAGCAAAATGTGCTACAAAAAAGGTCTTTCTCCATTAACAAATGGGTTCGGGGGGAAGGTACCAGGTATGGGACCACATCCACACCTTGTCTCATGCATGGTACTCTCTCTCCTTTTCCCAGTATGACTTCATGGAACGTCTGGACGGGAAGGAGAAGTGGAGTGTGGTTGAGTCTCCCAGGGAACGCCGGAGCATACAGACCTTGGTTCAGAATGAAGCCGTGTTTGTGCAGTACCTGGATGTGGGCCTGTGGCATCTGGCCTTCTACAATGATGG |
| --- | --- |
