# Supplementary material for: Identification of two unannotated miRNAs in classic Hodgkin lymphoma cell lines
Source: PLoS One. 2023 Mar 24;18(3):e0283186. doi: 10.1371/journal.pone.0283186 (PMC10038261; doi:10.1371/journal.pone.0283186)
Supplement: S4 Table — Genomic locations of amplified regions (GRCh38) and primer sequences used for bisulfite DNA pyrosequencing (DNA methylation analysis) of three regions corresponding to the two novel miRNA candidates. (DOCX) [file pone.0283186.s006.docx]

**Table S4. Bisulfite DNA pyrosequencing primers.** Genomic locations of amplified regions (GRCh38) and primer sequences used for bisulfite DNA pyrosequencing (DNA methylation analysis) of three regions corresponding to the two novel miRNA candidates.

| **miRNA candidate** | **Tested region/assay** | **Primer F 5' -> 3'** | **Primer R 5' -> 3'** | **Seqencing primer** | **Genomic location of amplified region (GRCh38)** |
| --- | --- | --- | --- | --- | --- |
| 2_nv_chr2_212678788 | I | ATGTGTAGTGTAAATGGAGGGGTAT | ATATCCCCACCCAAATCTCATCTT | AGAATAGTATGGGGGAA | chr2:212678797-212679018 |
| 3_nv_chr5_168090507 | I | AGTTAGGTTTGAGTTTTGGTATT | ATACAAAACTAACCAAAACTACATATTC | GTTTTTAGTTTTGATTTATGTTTT | chr5:167284647-167284800 |
|  | II | TGTGATTAATGGGATATGGGGTAGAGA | ACCAAAAAAATAACAACCATTTTATTAAATACCTA | TTTGAGAAATGGAGAAAATTTTA | chr5:168088403-168088609 |
