# Supplementary material for: Identification of two unannotated miRNAs in classic Hodgkin lymphoma cell lines
Source: PLoS One. 2023 Mar 24;18(3):e0283186. doi: 10.1371/journal.pone.0283186 (PMC10038261; doi:10.1371/journal.pone.0283186)
Supplement: S5 Table — pGEM ®-T Easy Vector primer sequences used for amplification and Sanger sequencing of nv_chr2_212678788 and 3_nv_chr5_168090507 novel miRNA sequences. (DOCX) [file pone.0283186.s007.docx]

**Table S5. pGEM ®-T Easy Vector primer sequences.** pGEM ®-T Easy Vector primer sequences used for amplification and Sanger sequencing of nv_chr2_212678788 and 3_nv_chr5_168090507 novel miRNA sequences.

|  | **M13 Forward** | **M13 Reverse** |
| --- | --- | --- |
| **Primer sequence 5’ -> 3’** | GTAAAACGACGGCCAG | CAGGAAACAGCTATGAC |
