# Supplementary material for: Identification of two unannotated miRNAs in classic Hodgkin lymphoma cell lines
Source: PLoS One. 2023 Mar 24;18(3):e0283186. doi: 10.1371/journal.pone.0283186 (PMC10038261; doi:10.1371/journal.pone.0283186)
Supplement: S7 Table — Functional enrichments found in the group of 45 genes that are the putative targets of the novel 2_nv_chr2_212678788 miRNA expressed in cHL cell lines (STRING, GO Consortium). (DOCX) [file pone.0283186.s009.docx]

**Table S7. Functional enrichment analysis results.** Functional enrichments found in the group of 45 genes that are the putative targets of the novel 2_nv_chr2_212678788 miRNA expressed in cHL cell lines (STRING, GO Consortium).

| **List of 45 genes used in functional enrichment analysis**  **(putative targets of novel** 2_nv_chr2_212678788**)** | *KCNN3, TEAD1, TECPR2, RBMS3, CLOCK, SPRED1, RORA, AKAP5, PDE10A, LRCH1, FNBP1L, JOSD1, DCAF6, FNIP1, FPGT, GABRA4, ZFHX3, BCL11A, GATA6, IKZF2, PDZD2, KLHL14, JAZF1, TMEM56, CDK6, MYCBP2, IDH1, ST8SIA1, AMMECR1L, NCOA2, PTAR1, DYRK1A, C1orf174, ZNF681, CCDC93, UNKL, NUDT16, SLC38A2, BRWD1, TEF, GTF2H1, FANCI, GRHL1, HIPK3, SLC17A6* |
| --- | --- |

| **Biological process** | **Term ID** | **FDR STRING** | **FDR GO Consortium** |
| --- | --- | --- | --- |
| Regulation of transcription by RNA polymerase II | GO:0006357 | 0.0130 | 2,55E-02 |
| Circadian rhythm | GO:0007623 | 0.0130 | 7,14E-03 |
| Positive regulation of gene expression | GO:0010628 | 0.0130 | 4,09E-02 |
| Circadian regulation of gene expression | GO:0032922 | 0.0130 | 6,86E-03 |
| Positive regulation of transcription, DNA-templated | GO:0045893 | 0.0130 | 3,52E-02 |
| Positive regulation of nucleobase-containing compound metabolic process | GO:0045935 | 0.0130 | 2,92E-02 |
| Rhythmic process | GO:0048511 | 0.0130 | 1,23E-02 |
| Positive regulation of RNA metabolic process | GO:0051254 | 0.0130 | 3,35E-02 |
| Positive regulation of macromolecule biosynthetic process | GO:0010557 | 0.0130 | 5,05E-02 |
| Positive regulation of nitrogen compound metabolic process | GO:0051173 | 0.0152 | 3,64E-02 |
| Regulation of RNA metabolic process | GO:0051252 | 0.0152 | 4,16E-02 |
| Positive regulation of cellular metabolic process | GO:0031325 | 0.0152 | 4,26E-02 |
| Positive regulation of macromolecule metabolic process | GO:0010604 | 0.0152 | 3,38E-02 |
